# Immunohistochemical Expression of Cyclooxygenase 2 (COX-2) as a Prognostic Marker and Its Correlation With Clinicopathological Parameters in Breast Cancer

**DOI:** 10.7759/cureus.65550

**Published:** 2024-07-27

**Authors:** Archana Perazhi Pulikkal, Mamatha K

**Affiliations:** 1 Pathology, Shri B. M. Patil Medical College Hospital and Research Centre, Bijapur Lingayat District Education (BLDE) (Deemed to be University), Vijayapura, IND

**Keywords:** er/pr, prognosis, vascular invasion, cox-2, breast cancer

## Abstract

Introduction

Breast cancer is considered the most common cancer among women. According to the literature, cyclooxygenase-2 (COX-2) expression in breast carcinoma is associated with aggressive tumor biology and acts as an independent prognostic marker. As COX-2 is a newly identified marker, studies are required to understand its immunoexpression and correlation with hormone receptor status and other prognostic factors, which helps in the therapeutic management of patients. Hence, this study evaluates the expression of COX-2 in breast carcinoma.

Methods

A hospital-based cross-sectional study was done on 55 mastectomy specimens collected at the Histopathology and Surgical Pathology Section of the Department of Pathology. The patient's age, histological type, tumor size, lymph node status, histological grade, and vascular invasion were noted. Immunohistochemical staining for estrogen receptor (ER), progesterone receptor (PR), human epidermal growth factor receptor 2/neu protooncogene (HER2/neu), and COX-2 markers was performed, and its results were compared with these clinicopathological and prognostic parameters. Results were subjected to statistical analysis.

Results

COX-2 expression was seen in 37 out of 55 cases (67.2%). Expression of COX-2 showed a statistically significant correlation with vascular invasion, ER-negative status, and PR-negative status. No statistical association was found between other parameters like age, tumor size, histological type, histological grade, lymph node status, and HER2/neu status.

Conclusion

The expression of COX-2 correlated strongly with well-established poor prognostic markers, such as vascular invasion, ER-negative status, and PR-negative status. Thus, expression of COX-2 suggests aggressive tumor biology, and it can be used as an independent prognostic marker.

## Introduction

Breast carcinoma is one of the most commonly diagnosed cancers and the most common cause of cancer death among women [1]. More than a million cases of breast carcinoma are diagnosed every year, accounting for over 23% of all malignancies that affect women globally [1]. According to GLOBOCAN 2022 data, breast cancer accounted for approximately two million cancer cases and six million cancer deaths, ranking second for incidence and fourth for mortality in the majority of world countries [2]. According to the World Health Organization (WHO) 2022, global estimations show dramatic differences in breast cancer risk based on human development. For instance, in countries with a very high Human Development Index (HDI), breast cancer affects one in 12 women in high-income countries over their lifetime, and one in 71 of them dies of it [2]. Breast cancer is a multifactorial disease caused by various genetic, hormonal, and environmental factors, with an interplay among the etiological factors and pathogenesis of breast cancer [3]. Based on gene expression profiling studies, breast carcinoma is classified into four categories: luminal A, luminal B, HER2 type, and basal-like/triple-negative [4]. These subtypes differ markedly in the prognosis and therapeutic targets they express [4]. Prognostic factors used in clinical oncology help select specific, individualized therapies and forecast the risk of tumor reappearance and metastasis [5]. Breast cancer patients have the worst prognosis because of the high rates of metastasis and local recurrence, which makes treatment ineffective [6]. The molecular subtyping of breast cancer significantly facilitates the accurate categorization of patients for the purpose of choosing the optimal treatment for customized breast cancer care, which results in distinct expression of biomarkers due to variations in DNA genetic makeup. Additionally, molecular characterization serves as a predictor of tumor aggressiveness and prognosis [7].

Cyclooxygenases (COXs) are fatty-acid oxygenases belonging to the myeloperoxidase superfamily. They are often referred to as prostaglandin H synthases or prostaglandin endoperoxide synthases. There are three isoforms: COX-1, COX-2, and COX-3 [8,9]. The COX group of enzymes is vital for converting arachidonic acid to prostaglandins. The metabolites generated from COX-2 could sustain the growth, invasion, metastatic dissemination, transformation, and premalignant hyperproliferation of the tumor [1,9]. In primary tumor cells, COX-2 and its byproducts, especially prostaglandin E2 (PGE2), promote carcinogenesis by acting through conventional cancer signaling pathways [1,9]. Because it is an induced enzyme, pro-inflammatory and mitogenic stimuli like growth factors and cytokines might influence its expression [9]. It also plays a vital role in estrogen regulation by producing PGE2, which enhances the expression of the cytochrome P450 enzyme complex, also known as aromatase, that catalyzes estrogen production, which is mediated through androgen [5]. Over-expression of COX-2 has been found in numerous carcinomas, including ovarian and breast cancer [1,9]. Patients with COX-2-positive malignancies exhibited far lower survival rates, and the tumors themselves appeared to be more aggressive [1,5,9]. After receiving treatment with COX-2 inhibitors that selectively inhibit COX-2 or non-selectively inhibit COX-2 expression with nonsteroidal anti-inflammatory drugs (NSAIDs), several studies have shown a decreased risk for breast, lung, prostate, and colon cancers [1,9]. More researches are needed to validate the potential role of COX-2 in the prognosis of breast cancer, as well as the protective effect of COX-2 inhibitors against breast cancer risk [8].

Nevertheless, the overall process underlying the clinical prognosis of patients with breast cancer remains unclear. Consequently, finding new prognostic indicators and a therapeutic focus are essential for the management [8]. Hence, this study aims to understand more about the immunohistochemical response of COX-2 in breast carcinoma and its prognostic importance.

## Materials and methods

This study was conducted on 55 patients who were diagnosed with invasive breast carcinoma from September 2022 to April 2024 and was conducted in the Histopathology Section, Pathology Department, Shri B. M. Patil Medical College Hospital and Research Centre, Bijapur Lingayat District Education (BLDE), Vijayapura. All modified radical mastectomy specimens of primary breast cancer received in the histopathology section were studied. Exclusion criteria included breast biopsy and lumpectomy specimens. Institutional ethical clearance was obtained for this study.

The tissue underwent standard processing after being stored in 10% buffered formalin. Sections with a thickness of four microns were cut from every tissue block. One section was stained with hematoxylin and eosin (H&E) for histopathological diagnosis, i.e., histopathological type (according to WHO classification) and histological grade (modified Scarff-Bloom-Richardson system of cancer grading). Another four sections from the same block were mounted on poly L lysine-coated slide from paraffin-embedded tissue blocks, which were subjected to estrogen receptor (ER)/progesterone receptor (PR), human epidermal growth factor receptor 2/neu protooncogene (HER2/neu) (according to the American Society of Clinical Oncology/College of American Pathologists (ASCO/CAP)), and COX-2 immunohistochemical staining. Immunohistochemical expression of COX-2 was correlated with prognostic factors such as the age of the patient, tumor size, tumor type, histological tumor grade, lymph node status, vascular invasion, ER, PR, and HER2/neu status. For the evaluation of COX-2, a predefined scoring system depending on the product of staining intensity and the percentage of positive tumor cells was used. Tumor cells with cytoplasmic positivity for COX-2 were considered.

COX-2 quantity score: 0 - no staining; 1 - 1%-10% cytoplasmic staining; 2 - 11%-50% cytoplasmic staining; 3 - 51%-80% cytoplasmic staining; 4 - ≥ 81% cytoplasmic staining

COX 2 staining intensity score: 0 - no staining;1 - weak staining; 2 - moderate staining; 3 - strong staining

COX-2 immunohistochemical score (IHS) is calculated by multiplying the quantity score by the staining intensity score: 0-3 = negative or faint staining; 4-8 = moderate/intermediate staining; 9-12 = strong/high staining. Intermediate and high-grade staining were considered positive.

The data were entered into a Microsoft Excel sheet (Microsoft® Corp., Redmond, WA), and statistical analysis was performed using JMP software (SAS Institute Inc., Cary, NC). Normally distributed continuous variables between the two groups were compared using an independent sample t-test. The Mann-Whitney U test was utilized for variables that were not regularly distributed. The chi-square test and Fisher’s exact test were used to compare categorical variables between the two groups. p < 0.05 was considered statistically significant. Two-tailed statistical tests were performed.

## Results

**Figure 1:**
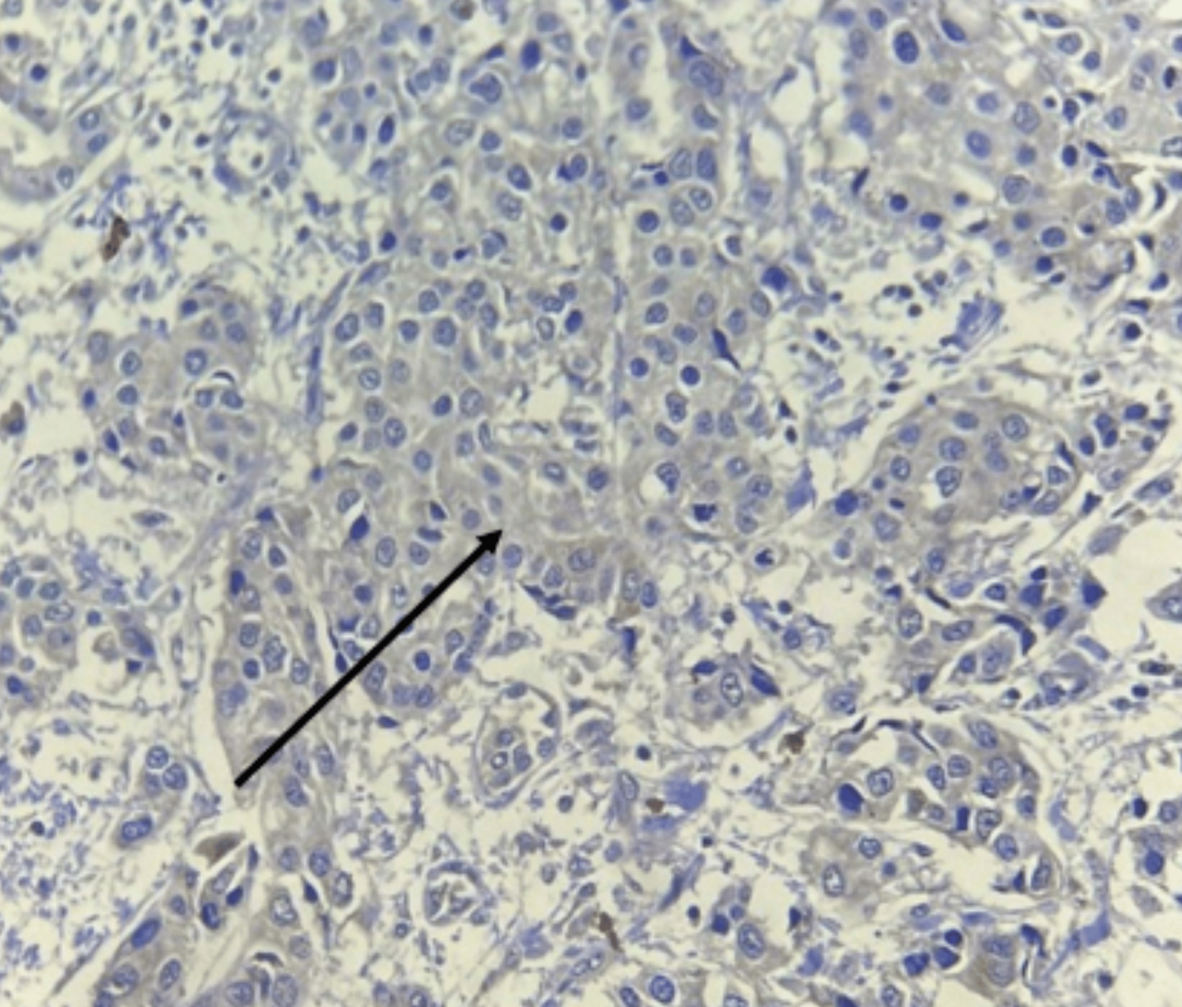
Microphotograph of immunohistochemical marker COX-2 showing cytoplasmic intermediate staining in infiltrating ductal carcinoma NOS (100×) COX-2, cyclooxygenase-2; NOS, not otherwise specified

**Figure 2:**
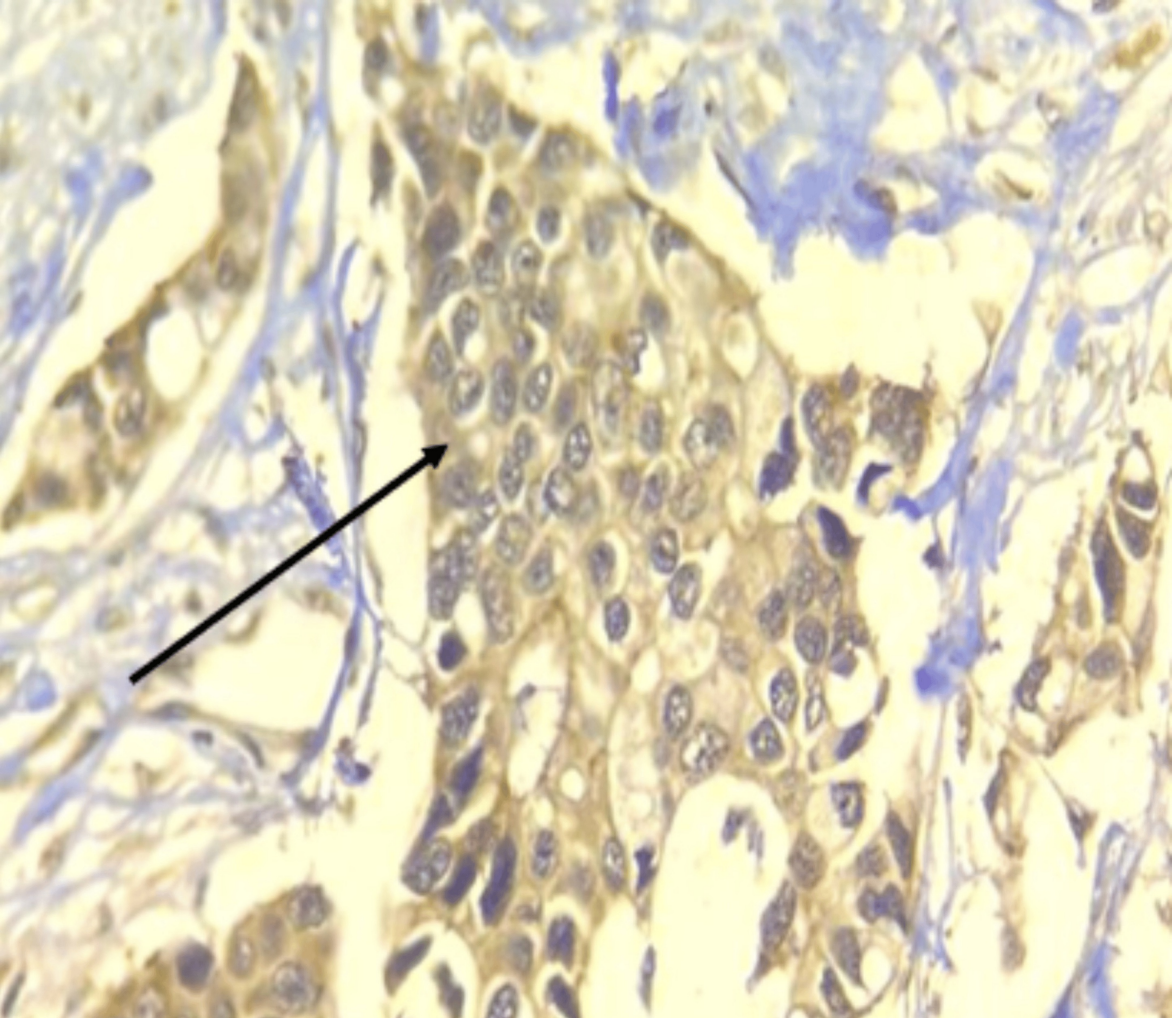
Microphotograph of immunohistochemical marker COX-2 showing cytoplasmic strong staining in infiltrating ductal carcinoma NOS (100×) COX-2, cyclooxygenase-2; NOS, not otherwise specified

The current study was conducted on 55 patients diagnosed with Invasive breast carcinoma. Staining status, pattern, and intensity of COX-2 expression were performed in malignant tumor cells. All positive cases showed cytoplasmic positivity. COX-2 expression was evaluated by quantity score. COX-2 IHSs with either intermediate staining, as shown in Figure 1, or intense staining, as shown in Figure 2, were considered positive. COX-2 positivity was seen in 37 (67.2%) cases, and in 18 (32.7%) cases, COX-2 was negative. The age group of the patients with Invasive breast carcinoma varied from 30 years to 80 years, with the mean age of the patients being 54.5 years and the median age being 55 years. The age group of 50 years and below accounted for 40% of the cases, and 60% were above 50 years.

The tumor ranged in size from 1 to 10 cm. Nineteen (51.40%) cases with COX-2 positivity fall into the group of tumor size T2, followed by eight (21.6%) cases that belonged to tumor size T1, which is followed by six (16.2%) cases of tumor size T3. The fewer cases with COX-2 positivity fall into tumor size T4, i.e., four cases (10.8%). Eight (44.4%) cases with COX-2 negative expression belonged to the group of tumor size T2, followed by five (27.8%) cases of tumor size T3, followed by four (22.2%) cases of tumor size T4. Only one (5.6%) case with negative COX-2 expression belonged to tumor size T1.

**Figure 3:**
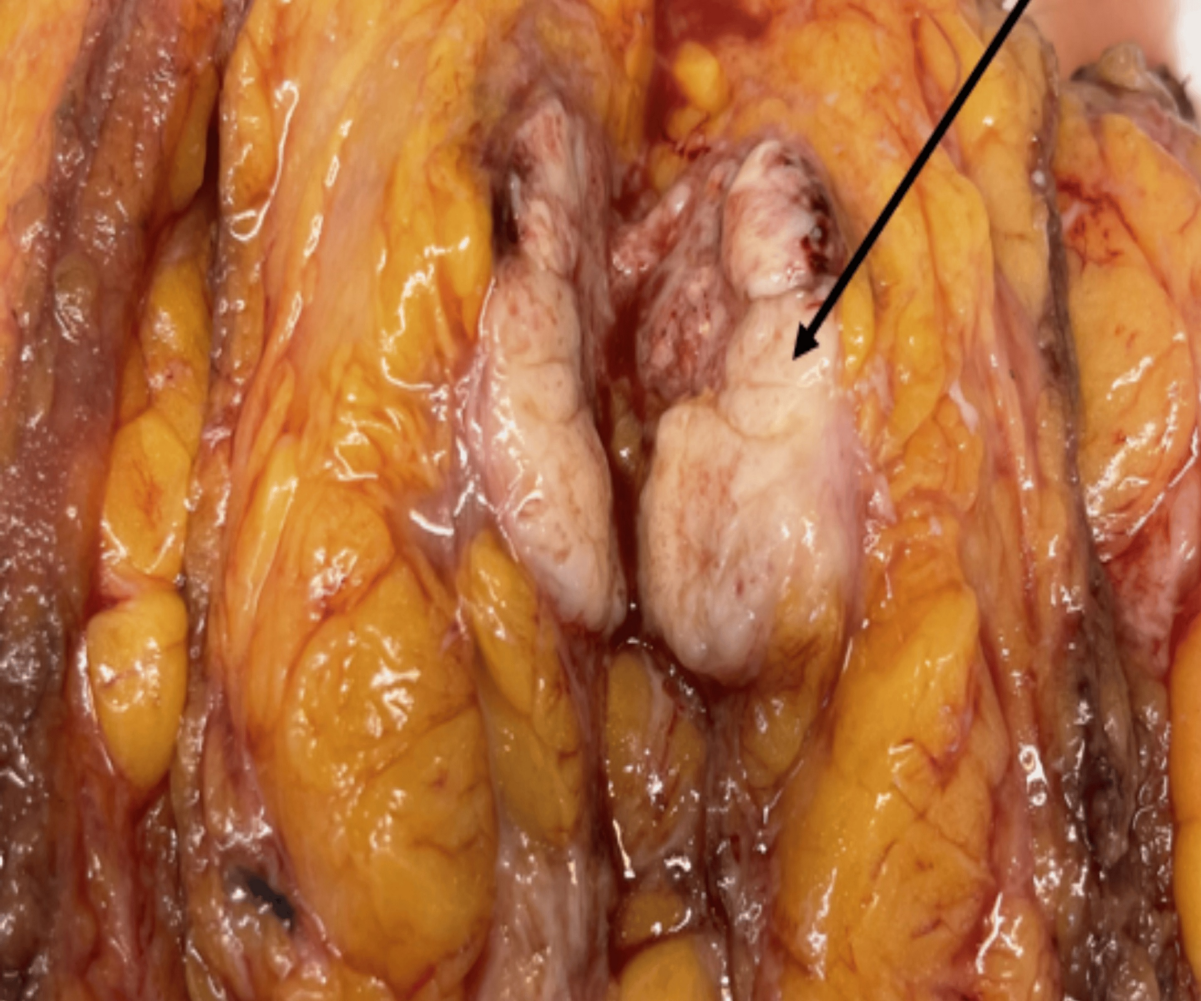
Macrophotograph showing a cut section of the tumor of infiltrating ductal carcinoma NOS NOS, not otherwise specified

**Figure 4:**
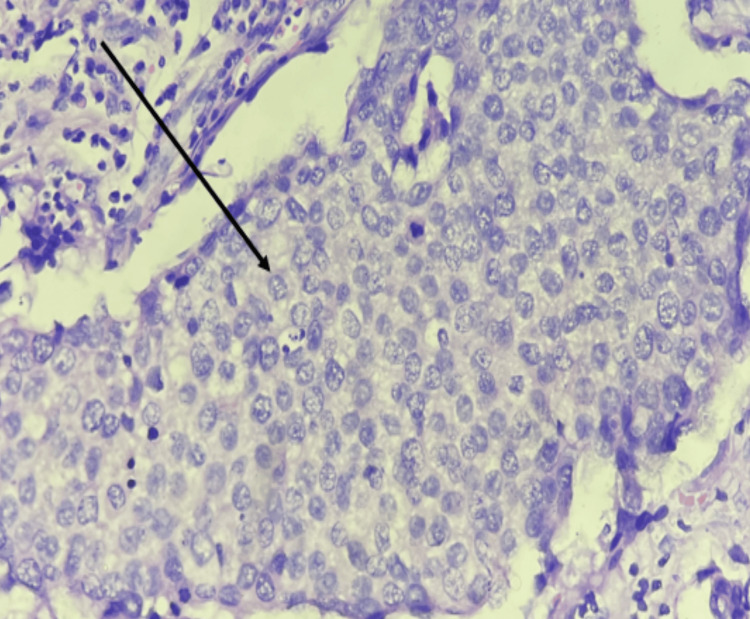
Microphotograph showing infiltrating ductal carcinoma NOS - Grade 3. Tumor cells arranged in sheets showing marked nuclear pleomorphism (H&E) (400×) H&E, hematoxylin and eosin; NOS, not otherwise specified

Among 51 cases of infiltrating ductal carcinoma-not otherwise specified (IDC-NOS), as shown in Figure 3 and Figure 4, 34 cases (91.9%) showed COX-2 positivity, and 17 cases (94.4%) showed COX-2 negativity. One case (2.7%) of invasive lobular carcinoma (ILC) showed COX-2 positivity. One case (2.7%) of encapsulated papillary carcinoma (EPC) showed COX-2 positivity. Among two cases of invasive papillary carcinoma (IPC), one case (2.7%) showed COX-2 positivity, and one case (5.6%) showed COX-2 negativity.

Most cases in this study with positive COX-2 expression belonged to Grade 2., i.e., 18 (48.6%) cases. This is followed by Grade 1, in which 11 (29.7%) cases showed COX-2 positivity, followed by eight (21.75) cases in Grade 3. Thirteen (72.2%) cases with negative COX-2 expression fall into Grade 2, followed by four (22.2%) cases of Grade 1 and one (5.6%) case of Grade 3.

Out of 55 cases of invasive breast carcinoma, 39 (70.9%) cases had positive nodal status; of these, 24 (64.9% ) were COX-2 positive, and 15 (83.3%) cases were COX-2 negative. Sixteen (29.1%) cases did not show lymph node metastases, of which 13 (35.1%) cases showed positive COX-2 expression.

**Figure 5:**
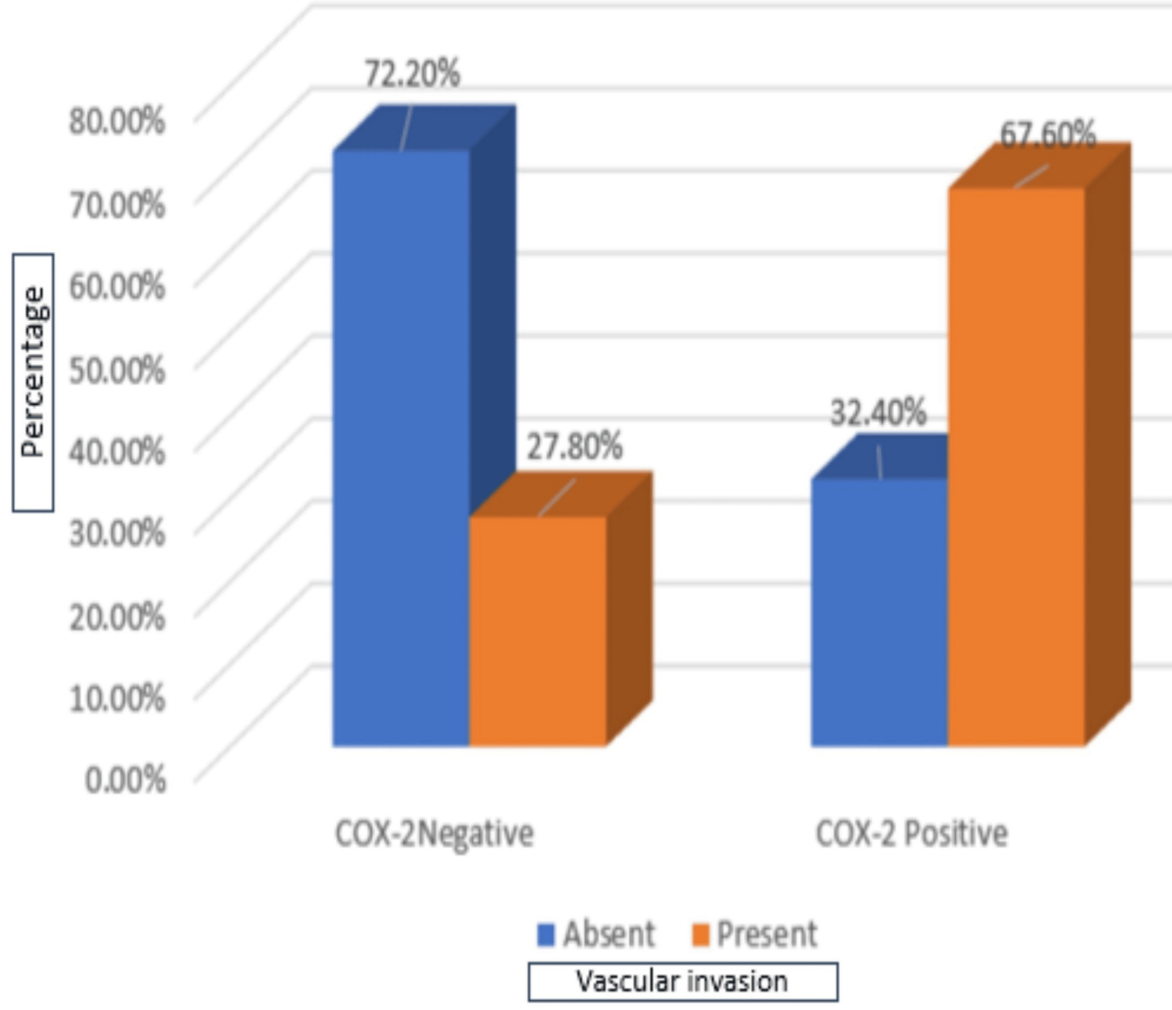
Graphical representation of COX-2 with vascular invasion COX-2, cyclooxygenase-2

Among 30 cases (54.5%) with vascular invasion, 25 (67.6%) cases showed COX-2 positivity, and five (27.8%) cases showed negative COX-2 expression. Among 25 cases without vascular invasion, 12 (32.4%) cases showed positive COX-2 expression, and 13 (72.2%) cases showed no COX-2 expression. Statistical significance between COX-2 and the presence of vascular invasion was found as the p-value was <0.05, as shown in Table 1 and Figure 5.

**Table 1:** Correlation of COX-2 expression with vascular invasion A p-value of 0.005 is considered significant. *Statistically significant. COX-2, cyclooxygenase-2

Vascular invasion	COX-2			Chi-square test	p-value
	Negative	Positive	Total		
Absent	13	12	25	7.732	0.005*
%	72.2%	32.4%	45.5%		
Present	5	25	30		
%	27.8%	67.6%	54.5%		
Total	18	37	55	-	
%	100.0%	100.0%	100.0%		

**Figure 6:**
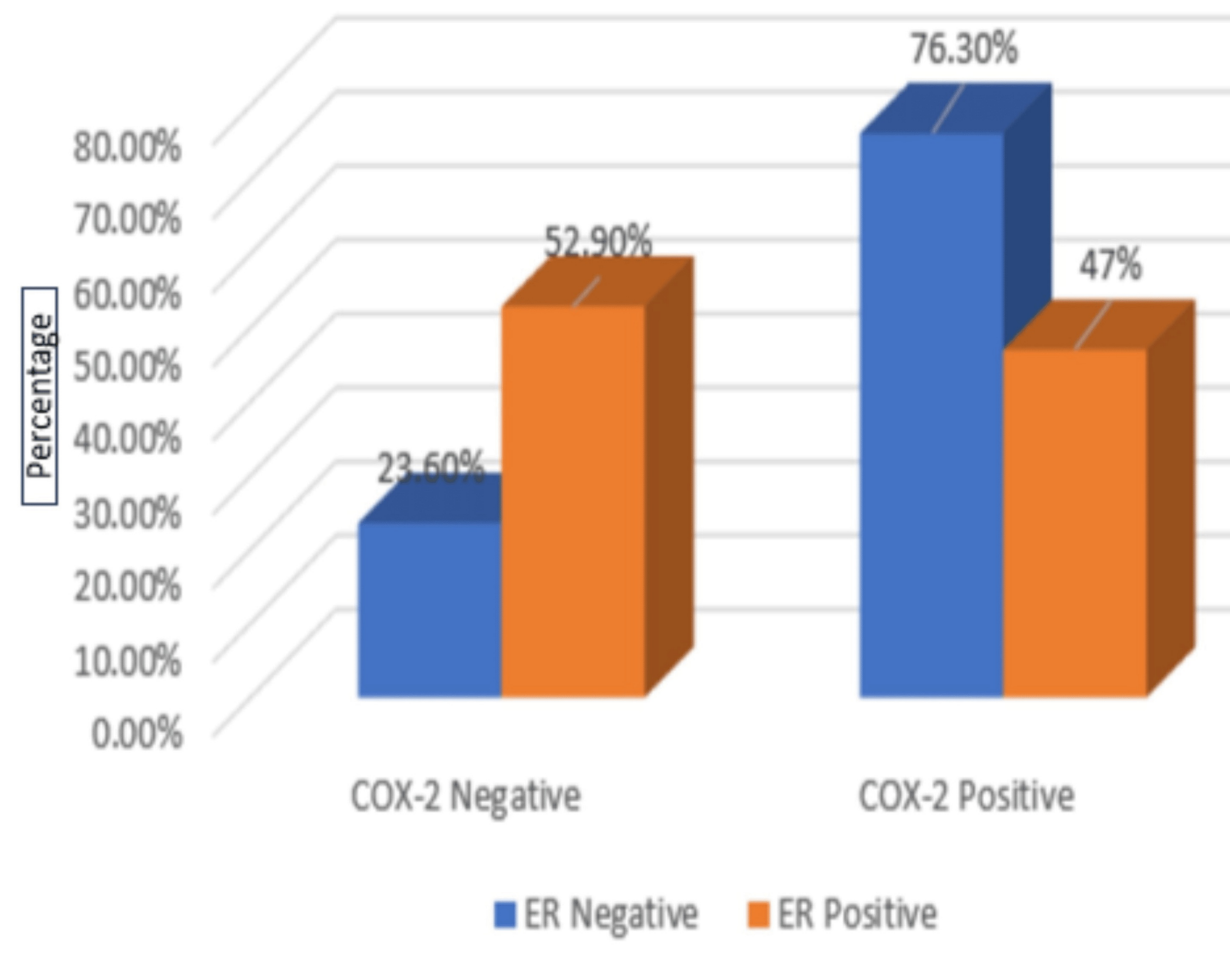
Graphical representation of COX-2 with ER expression COX-2, cyclooxygenase-2; ER, estrogen receptor

Of 38 ER-negative cases, 29 (76.3%) showed COX-2 positivity. Of 17 cases with positive ER expression, eight (47%) cases showed COX-2 expression. The p-value was 0.03, showing a statistically significant association between the expression of COX-2 and the negative ER status of the tumor, as shown in Table 2 and Figure 6.

**Table 2:** Correlation of COX-2 expression with ER receptor status A p-value of 0.03 was considered significant. *Statistically significant. COX-2, cyclooxygenase-2; ER, estrogen receptor

ER status	COX-2			Chi-square test	p-value
	Negative	Positive	Total		
Negative	9	29	38	4.56	0.03*
%	23.6%	76.3%	69.1%		
Positive	9	8	17		
%	52.9%	47%	30.9%		
Total	18	37	55	-	
%	100.0%	100.0%	100.0%		

**Figure 7:**
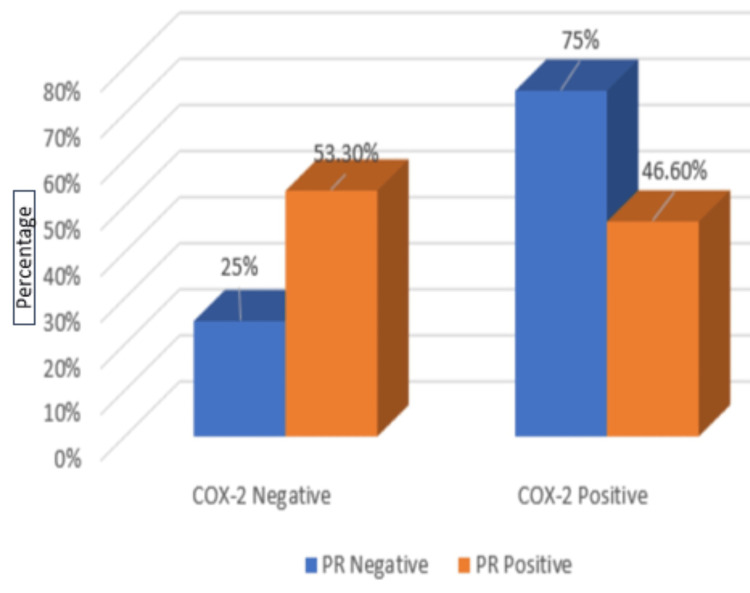
Graphical representation of COX-2 with PR expression COX-2, cyclooxygenase-2; PR, progesterone receptor

Of 40 PR negative cases, 30 (75%) cases showed COX-2 positivity. Of 15 cases with positive PR expression, seven (46.6%) cases showed COX-2 positivity; p-value was 0.04, showing a statistically significant association between the expression of COX-2 and the negative PR status of the tumor, as shown in Table 3 and Figure 7.

**Table 3:** Correlation of COX-2 expression with PR receptor status A p-value of 0.04 was considered significant. *Statistically significant. COX-2, cyclooxygenase-2; PR, progesterone receptor

PR status	COX-2			Chi-square test	p-value
	Negative	Positive	Total		
Negative	10	30	40	3.97	0.04*
%	25%	75%	72.7%		
Positive	8	7	15		
%	53.3%	46.6%	27.3%		
Total	18	37	55	-	
%	100.0%	100.0%	100.0%		

Of 30 HER2/neu negative cases, 17 (45.9%) cases showed COX-2 positivity. Of 24 cases with positive HER2/neu expression, 19 cases (51.4%) showed COX-2 positivity. No statistically significant correlation was found between the expression of COX-2 and other clinicopathological parameters, such as the age of the patient, tumor size, histological type, histological grade, lymph node status, and HER2/neu expression, as shown in Table 4.

**Table 4:** Comparison of COX-2 with various clinicopathological parameters p-values of 0.005, 0.03, and 0.04 were considered significant. COX-2, cyclooxygenase-2; EPC, encapsulated papillary carcinoma; ER, estrogen receptor; HER2/neu, human epidermal growth factor receptor 2/neu protooncogene; IDC-NOS, infiltrating ductal carcinoma-not otherwise specified; ILC, invasive lobular carcinoma; IPC, invasive papillary carcinoma; PR, progesterone receptor

Parameters	COX-2		Chi-square test	p-value
	Negative, no. of cases (%)	Positive, no. of cases (%)		
Age				
≤50 years	6 (33.3%)	16 (43.3%)	3.875	0.423
>50 years	12 (66.7%)	21 (56.7%)		
Tumor size				
T1	1 (5.6%)	8 (21.6%)	3.921	0.270
T2	8 (44.4%)	19 (51.4%)		
T3	5 (27.8%)	6 (16.2%)		
T4	4 (22.2%)	4 (10.8%)		
Histological type				
IDC-NOS	17 (94.4%)	34 (91.9%)	5.132	0.64
ILC	00	1 (2.7%)		
EPC	00	1 (2.7%)		
IPC	1 (5.6%)	1 (2.7%)		
Histological grade				
1	4 (22.2%)	11 (29.7%)	3.354	0.187
2	13 (72.2%)	18 (48.6%)		
3	1 (5.6%)	8 (21.6%)		
Lymph node status				
Involved	15 (83.3%)	24 (64.9%)	2.692	0.260
Not involved	3 (16.7%)	13 (35.1%)		
Vascular invasion				
Absent	13 (72.2%)	12 (32.4%)	7.732	0.005
Present	5 (27.8%)	25 (67.6%)		
ER status				
Negative	9 (23.6%)	29 (76.3%)	4.56	0.03
Positive	9 (52.9%)	8 (47%)		
PR status				
Negative	10 (25%)	30 (75%)	3.97	0.04
Positive	8 (53.3%)	7 (46.6%)		
HER2/neu				
Equivocal	00	1 (2.7%)	3.561	0.169
Negative	13 (72.2%)	17 (45.9%)		
Positive	5 (27.8%)	19 (51.4%)		

## Discussion

Breast cancer accounts for more than one million of the estimated 10 million neoplasms identified globally every year in both men and women, making it the most prevalent cause of cancer in females in both high- and low-resource settings [10,11]. More excellent knowledge of the molecular causes of metastatic disease would have applications in the medical field of diagnosis, treatment, and prognosis because metastatic disease is the cause of mortality linked to breast cancer [12]. In this particular context, the marker under discussion offered compelling evidence that the assessment and utilization of this marker for breast cancer will be essential for successful treatments that cause less harm to patients [13].

The mean age in this study was 54.5 years, and the median age was 55 years, meaning that postmenopausal patients had higher levels of COX-2 expression. However, no statistically significant association existed between COX-2 expression and the patient’s age. The same findings were found in the study [1,7] done by Nassar et al. and Solanki et al.

In the research [1] by Nassar et al., the maximum number of cases (36 out of 50 cases) belonged to sizes 2-5 cm and was not statistically significant. Similar findings were seen in the current study, where 19 cases (51.4%) with a tumor size of 2-5 cm (T2) showed COX-2 positivity. Nevertheless, our analysis found no evidence of a substantial relationship between COX-2 and tumor growth, as the p-value is 0.270. In other studies conducted by Solanki et al., Xu et al., Jana et al., Leyla et al., and Fatma et al., a strong relationship was found between COX-2 and larger tumor sizes with a p < 0.05 [14,15].

Histological types of invasive breast carcinoma included in our study were IDC-NOS, ILC, EPC, and IPC. Most cases were of IDC-NOS, i.e., 51 out of 55 cases (92.7%). However, none of the types were statistically significant with COX-2 expression. The same findings were noted in the study by Fatma et al., but no statistically significant association was found between COX-2 and histological type.

In the current research, Grade 2 tumors were found to have the highest percentage (56.4%). The same results were seen in the study [1,7] done by Nassar et al. and Solanki et al., where 37 out of 50 cases (74%) and 26 out of 50 cases (52%) were of Grade 2, respectively. However, no statistical significance was found between COX-2 and histological grade in our study (p = 0.187), which was concordant with the study conducted by these two authors. Research [5,14] done by Leyla et al. and Jana et al. showed that COX-2 expression was found to be associated with increased tumor grade, i.e., Grade 3, among the COX-2 positive group with a p < 0.05. They observed that higher expression of COX-2 in higher histologic grades implies that histological grade has a substantial prognostic value as a predictor of a poor prognosis.

Approximately 10-20% of women without axillary lymph node metastasis experience recurrence with distant metastasis [16,17]. In the present study, out of 39 cases with lymph node metastasis, 24 cases (64.9%) showed COX-2 positivity, which also revealed no statistically significant association between the expression of COX-2 and lymph node metastasis, as the p-value is 0.260. Studies done by Misron et al. and Jaudah et al. showed the same findings [18,19]. These findings were discordant with other studies conducted by Nassar et al., Leyla et al., Solanki et al., Xu et al., Jana et al., and Fatma et al., where there was a strong correlation between COX-2 expression and lymph node metastases, which may recommend that COX-2 overexpression is highly correlated with aggressive characteristics and unfavorable breast cancer prognostic factors. According to Jaudah et al., the majority of the tumors with distant metastases were detected with positive COX-2 staining (p = 0.003) [19].

Vascular invasion is an essential poor prognostic marker, and it aids in the risk factor for local recurrence [16,17]. In the current study, a strong statistically significant association was found between COX-2 and the presence of vascular invasion (p = 0.005). These results were correlated with a study done by Solanki et al., where vascular invasion was seen in 26 cases (89.7%) and not seen in three cases (10.3%) among COX-2 positive groups [7]. Also, in the study done by Jaudah et al., Mohammed et al., and Ameen et al., vascular invasion was found in 19 cases (90.4%), 14 cases (77.7%), and 90 cases (48.1%) among COX-2 positive groups and was found to be statistically significant with COX-2 expression [19-21]. Our study results were discordant with those of studies [1,8] conducted by Nassar et al. and Xu et al. Nassar et al. found 13 cases (81.3%) with vascular invasion showing COX-2 positivity and 30 cases (88.2%) without vascular invasion showing COX-2 positivity. However, the p-value was >0.05. Xu F et al. did a meta-analysis of 21 studies [8], including 6,739 patients with breast cancer, and it was concluded that COX-2 and vascular invasion were not statistically significant.

ER negative and PR negative status are poor prognostic markers. According to the current research, there is a strong significant association between COX-2 and negative ER, negative PR expression, and an insignificant association with HER2/neu expression. In the current study, COX-2 was statistically substantial with ER negative and PR negative status. These findings were concordant with the study [1,14] conducted by Nassar et al. and Jana D et al. In Nassar et al.'s study, 30 out of 50 (60%) cases showed ER-negative status, and 32 out of 50 (64%) cases showed PR-negative status among COX-2 positive groups, and the p-value was 0.001 and <0.001, respectively, which showed statistical significance. Jana D et al. also found that in ER-negative cell lines, COX-2 expression was related to mutated RAS, and reduced estrogen dependence in breast cells has been associated with increased COX-2 protein expression [14]. Also, PKC and mutated RAS have been related to a raised metastatic potential in cell lines [14]. Our findings were discordant with the research done by Leyla et al. They found statistical significance with COX-2 and positive ER expression. However, their study showed no statistical correlation between COX-2 expression, PR expression, and HER2/neu expression. Ristimaki et al. found that the upregulation of estrogen synthesis in ER-positive carcinoma may be the cause of COX-2 expression, which improves the conditions favorable for tumor cell growth [22]. Jana et al. also found that COX-2 was expressed more in positive HER2/neu tumors than negative HER2/neu tumors, as determined by the Western blot method and reverse transcription polymerase chain reaction (RT-PCR) technique. The p-value was <0.01 and was statistically significant [14].

Whereas in other studies conducted by Solanki et al., Xu et al., and Perez et al., there was no pathologically significant correlation between COX-2 and ER, PR, and HER2/neu status. Perez et al. also studied the correlation between COX-2 and other markers like Ki67 and Cytokeratin 5 [23]. However, the results were not statistically significant. To find out the source of heterogeneity among several studies, Xu et al. conducted a "meta reg" command using variables such as antibody catalog, detection method, publication date, and country [8]. The results demonstrated that no variables in the meta-regression contributed to the heterogeneity. In our study, COX-2 expression was found statistically significant in the vascular invasion, negative ER status, and negative PR status, which indicated the aggressive behavior of the tumor and poor prognosis. According to a study by Sahu et al., there were multiple levels of regulation for the widely reported increase of COX-2 in breast cancer. He also found that it is essential to the development of cancers because it increases the invasiveness of cells by mediating immune suppression by promoting the proliferation of epithelial cells, preventing apoptosis, promoting angiogenesis, and producing more mutagens [24].

Limitations of the study

Since there is heterogeneity in the expression of COX-2 with other parameters, a study with a larger size is required. Further studies with larger sample sizes will provide better insight into the COX-2 expression and its correlation with clinicopathological parameters.

Recommendations

Since COX-2 is a novel marker that can be used as a prognostic marker, further helping in evaluating the treatment options for patients with carcinoma breast, more studies are required with the precise predictive significance of COX-2 in relation to multiple additional factors that will help in the treatment of human breast cancer.

## Conclusions

In our study, COX-2 expression is strongly associated with poor prognostic factors in breast cancer, such as vascular invasion and ER-negative and PR-negative status. Overall expression of COX-2 in the study population was 67.2%. Among COX-2 positive groups, ER-negative cases were 76.3%, PR-negative cases were 75%, and cases with vascular invasion were 67.6%. This indicates that the expression of COX-2 is associated with the worst prognosis. However, no statistically significant correlation was obtained with other clinicopathological and prognostic parameters like age of the patient, tumor size, histological type, lymph node status, histological grade, and HER2/neu status. Since COX-2 expression shows aggressive tumor biology, it can be considered a prognostic marker that helps in future therapeutic studies using COX-2 inhibitors.
